# 常见炸药的稳定同位素比值分析方法研究进展

**DOI:** 10.3724/SP.J.1123.2020.09015

**Published:** 2021-04-08

**Authors:** Can HU, Hongcheng MEI, Hongling GUO, Zhenwen SUN, Zhanfang LIU, Jun ZHU

**Affiliations:** 公安部物证鉴定中心, 北京 100038; Institute of Forensic Science, Ministry of Public Security of China, Beijing 100038, China; 公安部物证鉴定中心, 北京 100038; Institute of Forensic Science, Ministry of Public Security of China, Beijing 100038, China; 公安部物证鉴定中心, 北京 100038; Institute of Forensic Science, Ministry of Public Security of China, Beijing 100038, China; 公安部物证鉴定中心, 北京 100038; Institute of Forensic Science, Ministry of Public Security of China, Beijing 100038, China; 公安部物证鉴定中心, 北京 100038; Institute of Forensic Science, Ministry of Public Security of China, Beijing 100038, China; 公安部物证鉴定中心, 北京 100038; Institute of Forensic Science, Ministry of Public Security of China, Beijing 100038, China

**Keywords:** 稳定同位素比值质谱, 同位素比值, 炸药, 溯源, stable isotope ratio mass spectrometry (IRMS), isotope ratio, explosive, traceability

## Abstract

炸药的深度比对与溯源对于爆炸案事件的侦破具有重大意义,以不同地域来源的原料或不同生产工艺生产的炸药,其组成元素的稳定同位素比值具有差异,因而稳定同位素比值可作为炸药深度比对与溯源的重要指标。稳定同位素比值质谱法(IRMS)作为一种高精度的稳定同位素比值测量手段,已逐渐发展成熟,与元素分析仪、气相色谱仪、液相色谱仪等仪器联用,在食品安全、环境保护、法庭科学等领域应用广泛。IRMS在炸药比对与溯源上亦发挥了重要作用,自1975年IRMS被应用于区分不同国家生产的三硝基甲苯(TNT)以来,IRMS已成功用于多种炸药的分析。但目前尚未见有文献系统地总结常见炸药的稳定同位素比值分析研究进展。该文介绍了稳定同位素比值分析的相关原理、仪器组成及特点,分别总结了硝酸铵、黑火药、TNT、太恩、黑索金等常见炸药的稳定同位素比值分析方法,汇总了文献报道的不同国家生产的硝酸铵、黑火药、TNT等炸药的稳定同位素比值。文章就不同炸药的稳定同位素比值差异、炸药生产、存储过程中相关因素对同位素比值的影响,爆炸前后稳定同位素比值的变化情况等内容进行了分析。本文还指出了目前炸药的稳定同位素比值分析研究中存在的问题,对可能的解决办法进行了讨论,对未来的发展方向提出了建议。

## Abstract (en)

The ratio of stable isotopes of the elements in explosives differs depending on the raw materials obtained from different geographical sources or the production processes adopted. Hence, this ratio can be used as an important index for the comparison and trace of explosives. Isotope ratio mass spectrometry (IRMS), a high-precision method for the analysis of stable isotope ratios, has evolved into a mature tool in this regard. In combination with elemental analysis, gas chromatography, liquid chromatography, etc., IRMS is widely used in food safety, environmental protection, forensic science, and other fields. IRMS also plays an important role in the comparison and trace of explosives. Since its application to distinguish trinitrotoluene (TNT) produced in different countries in 1975, IRMS has been successfully used in the analysis of various explosives. However, there is no systematic summary on the research progress on the stable isotope ratio analysis of common explosives. This paper provides a brief description of the related principle, instrumental composition, and characteristics of stable isotope ratio analysis. Methods for the stable isotope ratio analysis of common explosives such as ammonium nitrate, black powder, TNT, pentaerythritol tetranitrate (PETN), and cyclotrimethylene trinitroamine (RDX) are reviewed. The bulk stable isotopic ratio analysis method was used in most of the studies to determine the total isotope ratio of the sample. A compound-specific isotope analysis method was also employed to determine the isotope ratio of organic explosives in a complex matrix. The reported stable isotope ratios of explosives such as ammonium nitrate, black powder, and TNT produced in different countries are summarized. The discrimination ability of the stable isotope ratio for explosives is discussed. Based on the stable isotope ratio, explosives from different sources can be distinguished effectively. By combining the results of elemental analysis with the ICP-MS results, the discrimination efficiency of different samples could be further improved. The influence of relevant factors on the isotope ratio during the production and storage of explosives are collated. There is a strong correlation between the stable isotope ratios of explosives and raw materials. The stable isotope ratios of TNT, PETN, and other explosives are related to that of nitric acid used in the production. The stable isotope ratios of nitrogen and oxygen in the explosive are relatively stable and almost unchanged within one year of production. The complexity of the environmental matrix at the explosion site and the low concentration of explosive residues make the stable isotope analysis of explosive residues challenging. However, the changes in the stable isotope ratio before and after the explosion are discussed. Since there is no information on the application of stable isotope analysis to the traceability of explosives, the paper mentions that the standardized explosive sample pretreatment, stable isotope analysis method, collection and analysis of large amounts of explosive samples, and explosive stable isotope database are the basis of explosive traceability. This paper also outlines the existing challenges in the analysis of the stable isotope ratios of explosives, including the small number of explosive samples, lack of a stable isotope explosive analysis database, and difficulty in the stable isotope analysis of explosive residues. Possible solutions to these problems are proposed, followed by suggestions for the future development of the stable isotope ratio analysis of common explosives. The suggestions include establishing an effective extraction and enrichment method for explosive residues, combining IRMS with GC or LC for analyzing explosives, establishing a comprehensive process for the analysis of the stable isotope ratios of inorganic and organic explosives, and comparison and analysis of the stable isotope analysis data using statistical methods.

爆炸案件的快速侦破对打击犯罪、维护国家安全有重要意义。引起爆炸的物质从哪里来,是爆炸案件侦查过程中需要解决的重要问题之一。由于同位素分馏效应,不同地区的水、空气、植物等所含元素的稳定同位素比值具有一定的地域分布特性,因此稳定同位素比值可作为比对、溯源的依据^[1]^。稳定同位素比值质谱法(isotope ratio mass spectrometry, IRMS)作为一种新型、高精度的稳定同位素比值测量手段,已发展成熟并被广泛应用。在食品安全领域,用于酒精饮料^[2]^、蜂蜜^[3]^、食用油^[4]^等的产地溯源;在环境科学领域用于污染物来源推断^[5]^;在法庭科学领域用于大麻^[6]^、可卡因^[7]^、类固醇^[8]^等兴奋剂的来源推断,以及人类头发^[9]^、指甲^[10]^稳定同位素的生活区域分布特征研究。

以不同地域来源的原料或不同生产工艺生产的炸药,其稳定同位素比值同样具有差异。炸药的稳定同位素分析,可实现种类相同的炸药的进一步区分,为炸药的溯源提供依据,对爆炸类案件的迅速侦破、维护国家安全有重要意义。

在欧美法庭科学领域,IRMS已应用于硝酸铵、黑火药、三硝基甲苯(TNT)、太恩(季戊四醇四硝酸酯,PETN)、黑索金(环三亚甲基三硝胺,RDX)等炸药的分析^[11,12]^。但目前尚未见文献系统地总结IRMS在炸药分析上的应用。本文对稳定同位素比值质谱法的特点进行介绍,并全面地介绍了其在炸药分析上的应用,希望为从事相关研究的同行提供借鉴。

## 1 稳定同位素比值质谱法的特点

同位素分为稳定同位素和放射性同位素。其中,能够稳定存在、无放射性的同位素为稳定同位素,如^1^H和^2^H、^12^C和^13^C等。由于地质变化、气候变化及生物的活动和代谢等多种因素引起的同位素分馏效应,使得不同区域各元素的稳定同位素比例存在差异。

同位素比值*R*为某一元素的重同位素原子丰度与轻同位素原子丰度之比^[13]^,如^2^H/^1^H、^13^C/^12^C、^18^O/^16^O、^15^N/^14^N等。在自然界中,氢、碳、氮、氧、硫等轻元素的轻同位素相对丰度比重同位素高,因此*R*值通常很小,表述起来冗长繁琐。“*δ*”值为样品中某元素的同位素比值(*R*_sp_)与标准样品的同位素比值(*R*_st_)的相对偏差,如式(1)^[13]^所示:


(1) $\delta=\left(\frac{R_{\mathrm{sp}}}{R_{\mathrm{st}}-1}\right) \times 1 000 \‰$

在实际工作中常用“*δ*”值替代*R*值来标度同位素比值。*δ*值的大小与所采用的标准样品相关,因此在做同位素分析时首先要选择合适的标准样品。目前通用的同位素标准是由国际原子能委员会(IAEA)和美国国家标准和技术研究所(NIST)制定的^[14,15]^。

IRMS将扇形磁场仪与法拉第杯收集器相结合,可获得10^-4^~10^-6^的精密度,保障了自然丰度水平下同位素比值测定的准确性。与其他质谱一样,IRMS结构主要可分为进样系统、离子源、质量分析器、检测器以及真空系统等^[16]^。测量时,首先将样品转化成气体,然后在离子源中将气体分子离子化,离子化的气体因荷质比不同经扇形磁场分离,后经法拉第杯收集器收集检测。

依据分析对象的不同,稳定同位素分析技术可分为全样品稳定同位素分析(bulk stable isotope ratio analysis, BSIA)和特定化合物稳定同位素分析(compound-specific isotope analysis, CSIA)^[17]^。如图1a所示,BSIA一般与元素分析仪(EA)联用,样品经EA燃烧和还原后,转化为小分子气体,经GC分离后被导入IRMS,测定的是样品中所有化合物的总的同位素比值。CSIA一般与气相色谱仪(GC)或液相色谱仪(LC)^[18,19]^联用,样品经GC/LC分离后,单一组分经在线燃烧和还原后被导入IRMS,测定的是样品中单一组分的同位素比值(图1b)。GC/IRMS已广泛应用于食品安全、环境科学等领域复杂基质中特定化合物的分析。炸药的稳定同位素比值分析大多采用的BSIA方法,即将EA与IRMS联用,检测炸药中的总碳/氮/氢/氧的稳定同位素比值。

**图 1:**
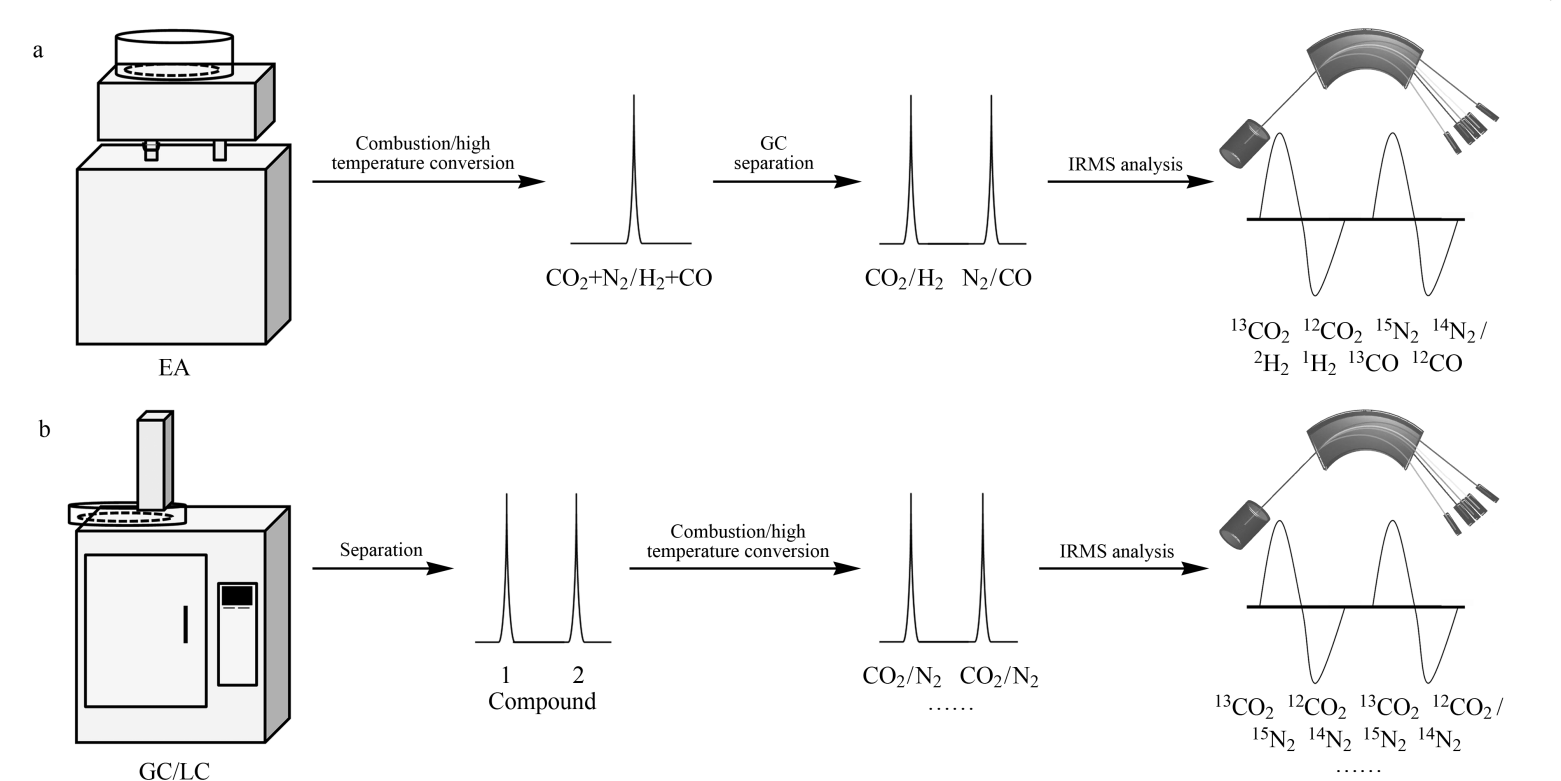
(a)BSIA与(b)CSIA的原理示意图

## 2 IRMS在炸药分析领域的应用

炸药的稳定同位素分析研究最早可追溯到1975年,Nissenbaum^[20]^采用双路进样同位素比值质谱(dual inlet isotope ratio mass spectrometry, DI-IRMS)分析了不同国家的TNT样品中碳的稳定同位素,实现了不同地区TNT样品的准确区分。随着技术的不断发展,IRMS被越来越多的应用于炸药的进一步区分与溯源。

### 2.1 无机炸药分析

2.1.1 硝酸铵

硝酸铵的生产有两种方式,一是转化法,即用硝酸钙与碳酸铵反应,产生硝酸铵和碳酸钙沉淀;另一种是中和法,即在硝酸中加入氨,中和至pH为7,得到硝酸铵,目前常用的是中和法。不同厂家的原料来源不同,氨由大气中的氢气和氮气反应产生,硝酸由氨氧化形成。不同地区大气中氮、氧稳定同位素比值不同,故而生产出的硝酸铵氮、氧稳定同位素比值亦有区别,可作为硝酸铵进一步区分与来源推断的依据。硝酸铵中含有氮(N)、氧(O)、氢(H)3种元素,由于硝酸铵易吸潮,铵根离子与大气中的水易发生氢交换,因此很难准确测定硝酸铵的^2^H/^1^H值^[21]^。氧交换的现象却很少发生,即使经过反复的吸水和脱水,*δ*^18^O值也基本不受影响^[22]^,因此氮、氧稳定同位素比值是硝酸铵分析的主要内容。

依据稳定同位素比值的不同区分不同硝酸铵样品是研究的重点。本研究小组采用EA/IRMS方法对产自北京、郑州、咸阳、长治、南京、柳州、荆门等不同地区的硝酸铵样品进行了分析,结果表明,结合*δ*^15^N、*δ*^18^O值可成功区分不同城市生产的硝酸铵样品。Brust等^[23]^对来自19个生产厂家的103个硝酸铵样品进行了EA/IRMS分析,并采用电感耦合等离子体质谱法对镁、钙、铁、锶在内的32种元素进行测定,基于线性判别分析(LDA)等统计方法对同位素分析结果和元素分析结果进行研判,成功实现了不同厂家硝酸铵样品的区分(LDA模型评价的敏感度和特异度分别为0.998和0.846),但对同一厂家不同批次的硝酸铵样品的区分效果却不佳。Benson等^[24]^对澳大利亚不同厂家生产的硝酸铵进行了稳定同位素分析,发现氮同位素对硝酸铵的区分能力有限,结合氧同位素和氢同位素分析结果可提高区分能力。

为了进一步提高对不同样品的区分能力,还有研究将硝酸铵中的铵根离子和硝酸根离子分离开,分别测定其同位素比值。Howa等^[25]^采用四苯硼酸钠与硝酸铵反应生成四苯基硼酸铵沉淀,将铵根离子从硝酸铵中分离开,分别测定铵根离子和硝酸根离子的氮稳定同位素,实现了42种硝酸铵样品的区分。但该文并未比较测定硝酸铵总氮的同位素比值对这42种硝酸铵样品的区分能力,未能体现分开测铵根离子和硝酸根离子氮稳定同位素的优势。Grimm等^[26]^在硝酸铵中加入氢氧化钾,将硝酸铵的铵根离子转换成氨气,剩下硝酸钾固体,从而将硝酸根离子从硝酸铵中分离开,然后分别测定其*δ*^15^N、*δ*^18^O值。文章比较了同一厂家2015年6月和11月两个时间生产的硝酸铵的总*δ*^15^N、*δ*^18^O值、硝酸根的*δ*^15^N值,结果都有显著差别,分析可能是生产所使用的硝酸原料来源不同导致的。

硝酸铵样品的保存对同位素分馏的影响也引起了关注。Gentile^[27]^分析了13份来自不同厂家不同批次的硝酸铵样品,发现样品在不同环境下存储一年对*δ*^15^N、*δ*^18^O值几乎没有影响。表1总结了文献报道的硝酸铵稳定同位素的分析情况。

**表 1:** 文献报道的硝酸铵稳定同位素分析情况

Number of samples	Sample source	*δ*^15^N			*δ*^18^O		Reference
		Reference materials	Measured value/‰		Reference materials	Measured value/‰	
103	Europe	IAEA-N-1, IAEA-N-2	-1.6-+4.8		USGS34, USGS35	+16.8-+24.8	[23]
13	Europe	IAEA-N-1, IAEA-N2, IAEA-NO-3	-6.1-+2.1		IAEA-CH-3	+16.5-+24.4	[27]
4	France	atmospheric N_2_	-2.4-+0.8		SMOW	+21.6-+23.3	[28]
2	Spain	atmospheric N_2_	-0.7-+2.5		SMOW	+18.0-+25.1	[29]

*SMOW: standard mean ocean water.

2.1.2 黑火药

黑火药是硝酸钾、木炭和硫黄的机械混合物。根据黑火药用途的不同,硝酸钾、木炭、硫黄3种成分的配比也有所不同,一般为75:15:10。不同厂家生产黑火药所用的原材料来源、配比和加工工艺不同,因此可通过稳定同位素分析实现区分。由于硫同位素分析过程中易残留、难去除,一般实验室较少开展相关检测,故而黑火药的稳定同位素分析通常包括氮、碳、氧3种元素。

Gentile等^[30]^对33个黑火药样品进行了EA/IRMS分析,发现生产厂家、批次、黑火药形态(粉末或粒状,粒径大小等)都会影响稳定同位素比值。该研究小组还考察了存储条件对同位素比值的影响,发现黑火药在正常环境下储存一年,其同位素比值不会发生明显改变。将黑火药暴露在高温高湿的环境下,对碳的同位素比值影响不大,却会显著影响氮的同位素比值。Lock^[31]^分析了从案件中收集的18种黑火药的*δ*^15^N、*δ*^18^O、*δ*^13^C值,虽然采用系统聚类和主成分分析方法对实验结果进行了分析,但由于样品本身的来源不明,很难对聚类结果进行评价。Mizota等^[32]^从日本6个不同地区的博物馆中收集了20种陈旧黑火药并进行了稳定同位素分析,发现这20种黑火药样品的*δ*^13^C值与柳树等C3植物的*δ*^13^C值相近,推断在日本生产木炭使用的主要为C3植物。表2总结了文献报道的黑火药稳定同位素分析情况,欧洲地区和日本地区所生产的黑火药的*δ*^15^N值差异明显。

**表 2:** 文献报道的黑火药稳定同位素分析情况

Number of samples	Sample source	*δ*^15^N			*δ*^18^O				*δ*^13^C			Reference
		Reference materials	Measured value/‰		Reference materials		Measured value/‰		Reference materials	Measured value/‰		
33	Switzerland, Germany, France	IAEA-N1, IAEA-N2, IAEA-N3	-28.0-+2.9		IAEA-CH-3	+15.0-+24.2			IAEA-USGS2, IAEA-CH-6, IAEA-CH7		-28.6--26.4	[27,30]
20	Japan	USGA-40	-0.7-+10.8		-	-			NBS-22		-28.3--25.9	[32]

-: not detected.

### 2.2 有机炸药分析

2.2.1 TNT

IRMS在炸药分析上最早的应用就是TNT^[20]^。TNT由甲苯与硝酸发生硝基反应而合成。因生产原料来源不同,或生产工艺的差异导致的同位素分馏效应不同,其稳定同位素比值亦存在差异。TNT的*δ*^15^N值与生产过程中所使用的硝酸相关,*δ*^13^C值与所使用的甲苯相关。Thermo公司于1995年通过*δ*^15^N、*δ*^13^C值实现了来自3个不同国家的TNT样品的区分^[33]^。Widory等^[28]^采用EA/IRMS对TNT、PETN、硝酸铵等炸药进行了分析,发现结合多个元素的稳定同位素比值可以提高不同炸药区分的准确度。该文章对1952-1992年不同年份生产的TNT稳定同位素比值进行了分析,发现不同年份生产的TNT其稳定同位素比值没有显著的差别,但文章没有考察储存条件对TNT样品稳定同位素比值的影响。表3总结了文献报道的TNT稳定同位素分析结果。

**表 3:** 文献报道的TNT稳定同位素分析情况

Number of samples	Sample source	Method	*δ*^15^N/‰	*δ*^18^O/‰	*δ*^13^C/‰	Reference
7	UK, USA, Israel, Italy, Canada,	DI-IRMS	-	-	-30.93--24.53	[20]
	Yugoslavia, Hungary					
14	Montenegrin, France	EA/IRMS	-8.7-+3.5	+16.5-+19.2	-29.9--23.5	[28]
5	USA, Croatia	GC/ITMS/IRMS	-5.36-+9.64	-	-26.42--22.21	[34]

DI: dual inlet; EA: elemental analysis; ITMS: ion trap mass spectrometry. -: not detected.

由于TNT毒性大,难降解,其残留物会对环境产生严重的不可逆的污染,因此TNT是环保领域十分关注的对象,稳定同位素分析同样是环保领域TNT污染物来源推断的一个有力手段。由于环境基质的复杂性,环保领域一般是采用CSIA法分析TNT,即将色谱与IRMS联用,将TNT与环境基质中的其他化合物分离开后特异性地检测TNT的同位素比值。而CSIA的不足在于灵敏度不高(对于单个化合物的稳定同位素分析,至少需要5 nmol C或10 nmol N)^[17]^,因此TNT的有效提取富集成为了关注的重点。Coffin等^[34]^通过固相萃取方法达到了99.8%的TNT回收率,后采用GC-离子肼-IRMS方法对TNT的稳定同位素进行分析,实现了5种不同来源的TNT样品的准确区分。作者还考察了固相萃取过程中TNT样品的稳定同位素分馏情况,结果并未发现明显的稳定同位素比值的变化。Berg等^[35]^采用固相微萃取方法对TNT进行提取富集,后通过GC-IRMS方法对*δ*^15^N、*δ*^13^C值进行了分析。文章系统地考察了方法的萃取效率、检出限、精密度等指标,并与EA/IRMS分析结果进行比较,两种方法的结果高度一致。

2.2.2 PETN

PETN是由季戊四醇(PE)与硝酸酯化而成。PETN的*δ*^15^N值与生产过程中所使用的硝酸相关,*δ*^13^C值与所使用的季戊四醇相关。

Benson等^[36]^对来自不同导爆索和助推器的15个PETN样品进行了初步的EA/IRMS分析,展示了将IRMS应用于区分不同PETN样品的良好前景。Widory等^[28]^对来自法国、黑山的12个PETN进行了EA/IRMS分析,结合ICP-MS元素分析的结果,实现了不同样品的准确区分。Howa等^[37]^对22个厂家的175个PETN样品进行了EA/IRMS分析,能够准确区分不同厂家生产的PETN。文章还研究了PETN与反应物PE和硝酸的同位素关系,结果表明PETN的*δ*^13^C值与反应物PE差别不大,而PETN中的*δ*^15^N值不仅与硝酸有关,还与反应条件有关。

2.2.3 其他有机炸药

TATP(三过氧化三丙酮)是以丙酮和双氧水为原料合成的。Bezemer等^[38]^通过TATP合成机理推导出TATP中的碳和氢源自丙酮,氧源自双氧水。通过EA/IRMS分析,发现丙酮与TATP的*δ*^13^C值之间存在线性关系。采用GC-IRMS对TATP进行分析,得到与EA/IRMS分析一致的结果。Benson等^[36]^比较了不同来源原料和不同反应条件下合成的TATP样品的*δ*^13^C、*δ*^18^O、*δ*^2^H值,通过3个同位素比值实现了18种不同条件下合成的TATP样品的区分。

RDX由六亚甲基四胺硝化合成。Lock等^[39]^用5种不同来源的六亚甲基四胺合成了5种RDX,并用EA/IRMS分别分析了六亚甲基四胺和RDX的*δ*^13^C、*δ*^15^N值,发现合成过程中存在同位素分馏效应,原料和产物RDX之间*δ*^13^C、*δ*^15^N值存在差异,且*δ*^15^N值差异更明显。Howa等^[40]^比较了Bachmann process和直接硝基化两种不同的合成方法下,合成原料和产物RDX稳定同位素比值的变化情况。相比直接硝基化,Bachmann process的*δ*^13^C、*δ*^15^N值变化更小,可能是合成过程中质量相关的同位素分馏引起的。Bernstein等^[41]^报道了在有氧和无氧生物降解过程中RDX的稳定同位素分馏。生物降解后的RDX经薄层色谱法纯化,然后进行EA/IRMS分析,发现在有氧环境下,*δ*^15^N、*δ*^18^O的富集因子分别为-2.1‰、-1.7‰,而无氧环境下分别为-5.0‰、-5.3‰。

六亚甲基三过氧化二胺(HMTD)也是以六亚甲基四胺为前体合成的。Lock等^[42]^同样采用IRMS对HMTD及其合成前体六亚甲基四胺进行了分析。比较了不同来源的六亚甲基四胺及其合成的HMTD的*δ*^13^C、*δ*^15^N值,发现同位素分馏程度受反应效率的影响,产率越高,生成的HMTD的稳定同位素比值与前体材料更接近。

塞姆汀H型炸药(Semtex-H),一种塑胶型炸药,由增塑剂(约20%的聚合物基质和脂肪烃)和约80%的不同比例的PETN和RDX组成。Pierrini等^[43]^采用EA/IRMS测定了26种Semtex-H的总*δ*^13^C、*δ*^15^N值,并用似然比的方法对分析结果进行了评价。

赤藓糖醇四硝酸酯(ETN)由赤藓糖醇和硝酸盐合成。Bezemer等^[44]^通过IRMS比较了合成前体与产物ETN的稳定同位素比值,发现前体与产物的同位素比值存在线性关系。文章还发现熔铸的ETN样品与存放了2年的陈旧ETN样品之间稳定同位素比值无明显差异,说明ETN的稳定同位素比值具有很好的稳定性。

### 2.3 爆炸后的炸药残留物稳定同位素分析

实际爆炸案事件中遇到的更多的是炸后的残留物,但目前爆炸残留物的稳定同位素分析开展的较少。原因在于爆炸现场往往较为复杂,由多种物质混合而成,会干扰稳定同位素的分析,且爆炸残留物的量往往较低,给检测带来了挑战。McGuire等^[45]^率先报道了爆炸前后TNT、奥克托今(HMX)、三氨基三硝基苯(TATB)和3个塑胶炸药的同位素比值变化情况。结果除了TNT的*δ*^13^C值基本没变以外,其他炸药在爆炸过程中都发生了同位素分馏,*δ*^2^H、*δ*^15^N值均发生改变。Benson等^[24]^引爆了6种硝酸铵炸药,研究了爆炸前后的*δ*^15^N值的变化,发现爆炸后存在显著的*δ*^15^N的富集,文章对同位素富集产生的原因进行了探讨。但这两篇文章都是在自己布置的环境中模拟爆炸实验,爆炸残留物的收集相对容易,减少了环境基质的干扰。

### 2.4 炸药的溯源

炸药的稳定同位素分析一方面可以增加炸药分析的维度,实现种类相同的炸药的进一步比对,另一方面可以为炸药的溯源奠定基础。未知来源炸药的溯源,对于案件的侦破具有重要意义,然而,目前并没有文献报道稳定同位素分析在炸药溯源上的应用。其挑战在于,一是标准、规范化的炸药样品前处理、稳定同位素分析方法有待建立,二是大量炸药样本的采集与分析,以及在此基础上的炸药稳定同位素数据库有待建立。炸药稳定同位素分析技术的不断成熟为溯源工作奠定了较好的基础。

## 3 结论

针对常见无机、有机炸药的稳定同位素分析已经开展了大量研究,充分验证了通过稳定同位素比值特征实现成分相同的炸药的进一步区分与溯源的可行性。目前的研究主要聚焦在:(1)不同厂家、批次生产的炸药的区分;(2)合成原料、炸药产物的稳定同位素比值之间的关系,合成条件对炸药产物的稳定同位素比值的影响;(3)储存条件对炸药稳定同位素比值的影响;(4)爆炸前后稳定同位素比值的变化等。尽管已经涉及炸药生产、存储、使用的诸多方面,但还存在以下问题:(1)样品来源问题。研究或是涉及的炸药种类较少,远达不到溯源的要求,相关数据库还有待建立;或是炸药来源不清,无法对分析结果给出进一步解释。(2)目前的研究大多注重不同来源样品之间的区分,缺少对样品本身均一性的考察。(3)目前的研究主要聚焦在爆炸前炸药原药的分析,爆炸残留物的稳定同位素检测仍然是个挑战。

针对炸药的稳定同位素分析,未来可能的发展方向有:一是建立复杂基质中微量爆炸残留物的检测方法,爆炸残留物的有效提取、富集,IRMS与色谱的联用以分离爆炸残留物与其他物质,是可能的解决办法;二是建立无机、有机炸药稳定同位素比值分析综合流程,在实际案件中有可能碰到无机、有机炸药混用的情况,有必要建立一套综合分析流程;三是炸药的溯源分析,这一方面依赖于大量样本的分析与数据库的建立,一方面需充分结合统计学方法进行稳定同位素分析数据的比较与分析。
